# 第八版国际肺癌TNM分期修订稿解读

**DOI:** 10.3779/j.issn.1009-3419.2016.06.07

**Published:** 2016-06-20

**Authors:** 波 叶, 珩 赵

**Affiliations:** 200030 上海，上海交通大学附属胸科医院胸外科 Department of Thoracic Surgery, Shanghai Chest Hospital, Shanghai Jiaotong University, Shanghai 200030, China

**Keywords:** 肺肿瘤, TNM分期, 诊断, 治疗, Lung neoplasms, TNM Stage, Diagnosis, Treatment

## Abstract

目前临床使用的肺癌分期是国际抗癌联盟（Union for International Cancer Control, UICC）于2009年1月颁布的第七版TNM分期。近年来，随着肺癌诊断技术的提高以及个体化治疗、分子靶向治疗等精准治疗模式的改变，肺癌的生存率及预后也有了明显的提高，旧的分期标准已难以适应当前的快速发展的临床需求。因此国际肺癌研究学会（International Association for the Study of Lung Cancer, IASLC）2015年对肺癌分期系统进行了更新，其修订稿发表于《*Journal of Thoracic Oncology*》，新版分期计划于2017年1月正式颁布实施。新分期标准采纳了来自16个国家的35个数据库，包含了自1999年-2010年间新发病的94, 708例肺癌病例。新版分期的优势在于能够更好的显示患者的预后，对临床具有更高的指导价值。

## Abstract (en)

The currently adopted staging system for lung cancer is the seventh edition of the TNM staging edited by Union for International Cancer Control (UICC) in January, 2009. In recent years, with the advances of techniques in lung cancer diagnosis and the treatment trends towards precision treatment modalities such as individualized therapy and molecular targeted therapy, the survival and prognosis of lung cancer has been significantly improved. The old staging standard is difficult to satisfy the currentrapidly developing clinical needs. Therefore, the International Lung Cancer Research Society (International Association for the Study of Lung Cancer, IASLC) updated the stage of lung cancer in 2015, and the forthcoming eighth edition of the TNM Classification for Lung Cancer, which will be formally adopted in Jan. 2017, has been published in *Journal of Thoracic Oncology*. The new staging system has adopted 35 databases from 16 countries, including 94, 708 cases treated between 1999 and 2010. The advantages of the new staging lies in its higher prognosis prediction and clinical guidance value.

目前世界各国临床应用的国际抗癌联盟（Union for International Cancer Control, UICC）TNM分期的目的是为了更好的指导临床患者的预后，此次第八版肺癌TNM分期较第七版分期能够更好的反映不同分期患者的预后。新的第八版肺癌分期修订稿已发表于《*Journal of Thoracic Oncology*》^[1-8]^，并于2017年1月正式开始实施，本文对第八版分期修订稿的具体内容进行详细解读。

## 1 数据来源

国际协会肺癌研究会（International Association for the Study of Lung Cancer, IASLC）在1990年-2000年间评估了大约有8.1万名确诊为肺癌患者，制定了第七版修订的肺癌TNM分期^[9-13]^。而第八版肺癌TNM分期是IASLC的数据库在1999年和2010年之间搜集了94, 708新确诊的肺癌患者，这些患者来源于16个国家的35个中心。其中欧洲贡献了46, 560例患者，亚洲41, 705例患者，北美4, 660例患者，澳大利亚1, 593例患者和南美190例患者。经过筛选后77, 156例（70, 967例非小细胞肺癌和6, 189例小细胞肺癌）进行分析。主要是针对预后进行分析，从而制定TNM分期。其中第七版分期完全是建立在回顾性的数据基础上的，而第八版的数据则克服了这点，在已经建立的TNM分期基础上进行前瞻性的分析，形成的第八版分期对于预后更加有指导作用。

## 2 第八版TNM分期变动的主要内容

在第七版分期的基础上，第八版分期能够更好地指导预后和临床治疗，表 1和表 2详述了新的第八版TNM分期，表 3比较了第六、七、八版TNM分期的内容。

**1:** International Association for the Study of Lung Cancer（IASLC）第八版TNM分期修订稿 International Association for the Study of Lung Cancer (IASLC) Eighth Edition of the TNM Classification for Lung Cancer

T分期：
Tx：未发现原发肿瘤，或者通过痰细胞学或支气管灌洗发现癌细胞，但影像学及支气管镜无法发现。
T0：无原发肿瘤的证据。
Tis：原位癌。
T1：肿瘤最大径≤3 cm，周围包绕肺组织及脏层胸膜，支气管镜见肿瘤侵及叶支气管，未侵及主支气管。
T1a（mi）：微浸润腺癌（minimally invasive adenocarcinoma, MIA）；^a^
T1a：肿瘤最大径≤1 cm；^b^
T1b：肿瘤最大径>1 cm，≤2 cm；
T1c：肿瘤最大径>2 cm，≤3 cm；
T2：肿瘤最大径>3 cm，≤5 cm；侵犯主支气管（不常见的表浅扩散型肿瘤，不论体积大小，侵犯限于支气管壁时，虽可能侵犯主支气管，仍为T1），但未侵及隆突；侵及脏层胸膜；有阻塞性肺炎或者部分或全肺肺不张。符合以上任何一个条件即归为T2。
T2a：肿瘤最大径>3 cm，≤4 cm；
T2b：肿瘤最大径>4 cm，≤5 cm；
T3：肿瘤最大径>5 cm，≤7 cm，直接侵犯以下任何一个器官，包括：胸壁(包含肺上沟瘤)、膈神经、心包；同一肺叶出现孤立性癌结节。符合以上任何一个条件即归为T3；
T4：肿瘤最大径>7 cm；无论大小，侵及以下任何一个器官，包括：纵隔、心脏、大血管、隆突、喉返神经、主气管、食管、椎体、膈肌；同侧不同肺叶内孤立癌结节。
N分期
Nx：区域淋巴结无法评估。
N0：无区域淋巴结转移。
N1：同侧支气管周围及（或）同侧肺门淋巴结以及肺内淋巴结有转移，包括直接侵犯而累及的。
N2：同侧纵隔内及（或）隆突下淋巴结转移。
N3：对侧纵隔、对侧肺门、同侧或对侧前斜角肌及锁骨上淋巴结转移。
M分期
M0：无远处转移。
M1：远处转移。
M1a：局限于胸腔内，包括胸膜播散（恶性胸腔积液、心包积液或胸膜结节）以及对侧肺叶出现癌结节（许多肺癌胸腔积液是由肿瘤引起的，少数患者胸液多次细胞学检查阴性，既不是血性也不是渗液，如果各种因素和临床判断认为渗液和肿瘤无关，那么不应该把胸腔积液纳入分期因素）。^c^
Mlb：远处器官单发转移灶为M1b。^d^
Mlc：多个或单个器官多处转移为M1c。
^a^: 单发结节，肿瘤直径≤3 cm，贴壁生长为主，病灶中任何一个浸润灶的最大直径≤5 cm。 ^b^: 任何大小的非常见浅表肿瘤，只要局限于支气管壁，即使累计主气管，也定义为T1a。 ^c^: 大部分肺癌患者胸腔积液或者心包积液是由肿瘤所引起的，但是如果胸腔积液多次细胞学未能找到癌细胞，胸腔积液又是非血性和非渗出的，临床判断胸腔积液和肿瘤无关，属于M0。 ^d^: 具有这些特点的T2肿瘤，如果≤4 cm或者直径不能确定的属于T1a，如果>4 cm，≤5 cm归T2b。

**2:** 第八版TNM分期 Eighth edition of the TNM classification

	N0	N1	N2	N3	M1a	M1b	M1c
T1a	Ⅰa1	Ⅱb	Ⅲa	Ⅲb	Ⅳa	Ⅳa	Ⅳb
T1b	Ⅰa2	Ⅱb	Ⅲa	Ⅲb	Ⅳa	Ⅳa	Ⅳb
T1c	Ⅰa3	Ⅱb	Ⅲa	Ⅲb	Ⅳa	Ⅳa	Ⅳb
T2a	Ⅰb	Ⅱb	Ⅲa	Ⅲb	Ⅳa	Ⅳa	Ⅳb
T2b	Ⅱa	Ⅱb	Ⅲa	Ⅲb	Ⅳa	Ⅳa	Ⅳb
T3	Ⅱb	Ⅲa	Ⅲb	Ⅲc	Ⅳa	Ⅳa	Ⅳb
T4	Ⅲa	Ⅲa	Ⅲb	Ⅲc	Ⅳa	Ⅳa	Ⅳb

**3:** 肺癌第六、七、八版TNM分期比较 IASLC-sixth, seventh, eighth edition of the TNM classification for lung cancer

TNM分期	第六版	第七版	第八版
肿瘤直径≤1 cm	T1	T1a	T1a
肿瘤直径>1 cm，≤2 cm	T1	T1a	T1b
肿瘤直径>2 cm，≤3 cm	T1	T1b	T1c
肿瘤直径>3 cm，≤5 cm	T2	T2a	T2a（>3 cm至≤4 cm）,T2b（>4 cm至≤5 cm）
肿瘤直径>5 cm，≤7 cm	T2	T2b	T3
肿瘤直径>7 cm	T2	T3	T4
支气管受累距隆突 < 2 cm，但不侵犯隆突，和伴有肺不张/肺炎	T3	T3	T2
侵犯膈肌	T3	T3	T4
同肺叶内其他肺结节	T4	T3	①Mla局限于胸腔内，包括胸膜播散（恶性胸腔积液、心包积液或胸膜结节）以及对侧肺叶出现癌结节归为Mla；②远处器官单发转移灶为M1b；③多个或单个器官多处转移为M1c。
在同一侧其他肺叶结节	M1	T4	
胸膜播散（包括恶性胸腔积液及孤立胸膜结节）	T4	M1a	
心包播散（包括恶性心包腔积液及孤立心包结节）		M1a	
胸腔内转移	M1	M1a	
胸腔外转移	M1	M1b	
T2bN0M0	Ib	Ⅱa	
T2aN1M0	Ⅱb	Ⅱa	
T4N0-1M0	Ⅲb	Ⅲa	
IA期		T1a，T1b	T1a，T1b，T1c
T3N1M0		Ⅱb	Ⅲa
T3N2		Ⅲa	Ⅲb
T3-4N3		Ⅲb	Ⅲc
M分期			M1a和M1b更新为Ⅳa， M1c更新为Ⅳb

### 2.1 T分期

① 将T1细分为T1a（≤1 cm），T1b（> 1 cm至≤2 cm），T1c（> 2 cm至≤ 3 cm）；② T2细分为T2a（> 3 cm至≤4 cm）和T2b（> 4 cm至≤5 cm）；③重新分类 > 5 cm且≤7 cm的肿瘤分为T3；④重新分类超过7 cm或更大的肿瘤为T4；⑤支气管受累距隆突 < 2 cm，但不侵犯隆突，和伴有肺不张/肺炎则归为T2；⑥侵犯膈肌分为T4；⑦删除纵隔胸膜浸润这一T分期术语。

### 2.2 N分期

继续使用原N分期方法。但提出了转移淋巴结的位置：nN（单站与多站），存在和不存在跳跃式淋巴结转移，pN1a，pN1b，pN2a1，pN2a2和pN2b可能对预后的评价更为精确。

### 2.3 M分期

将M分期进一步细分为Mla，M1b和M1c，其中M1a与第七版定义一致，将M1b也细分，单器官转移独列为新的M1b，具体如下：①Mla局限于胸腔内，包括胸膜播散（恶性胸腔积液、心包积液或胸膜结节）以及对侧肺叶出现癌结节归为Mla；②远处器官单发转移灶为M1b；③多个或单个器官多处转移为M1c。

### 2.4 TNM分期

①Ⅰa期分为Ⅰa1，Ⅰa2和Ⅰa3；②T1a, bN1由Ⅱa期改为Ⅱb期；③T3N1由Ⅱb期改为Ⅲa期；④T3N2由Ⅲa期改为Ⅲb期；⑤T3-4N3更新为Ⅲc期；⑥M1a和M1b更新为Ⅳa，M1c更新为Ⅳb。

## 3 第八版TNM分期修改部分的解读

### 3.1 T分期

#### 3.1.1 突出了肿瘤大小对预后的影响

简单的来说，新的分期更加重视了肿瘤大小对于预后的影响。研究^[2]^发现肿瘤大小是影响肺癌患者预后的重要因素。原发肿瘤直径≤1 cm，1 cm-2 cm，2 cm-3 cm，3 cm-4 cm，4 cm-5 cm，5 cm-6 cm，6 cm-7 cm这7个组别，其预后根据肿瘤的大小预后不同，肿瘤越大，预后越差。对于那些直径≤5 cm的患者，肿瘤至今每增加1 cm，其预后明显下降（*P* < 0.001），而对于肿瘤最大径 > 5 cm，≤7 cm的患者生存率变化不大，因此将其统称为T3。由于肿瘤最大径≤3 cm及 > 3 cm生存差异很大（*P* < 0.001），因此将3 cm仍作为T1、T2的分界点，前三组T1又依次分为T1a、T1b、T1c，中间两组T2分又为T2a及T2b，每个分期间隔为1 cm。同时研究发现肿瘤最大径 > 7 cm患者预后与七版分期的T4患者生存率类似，因此新版将 > 7 cm归为T4。

#### 3.1.2 主支气管受累距隆突的距离不再作为T分期的依据

第七版分期中将肿瘤累及主支气管距离隆突≥2 cm归为T2，累及主支气管且距离隆突 < 2 cm但未累及隆突者为T3。而研究却发现，在所有的研究人群中，累及主支气管且距离隆突≥2 cm与其他因素T2预后一致，生存差异并无统计学意义，而累及主支气管且距离隆突 < 2 cm但未累及隆突者，预后明显好于其他因素T3，因此新版分期对于主支气管受累，只要未侵犯隆突，无论距离隆突多远均归为T2。

#### 3.1.3 肺不张/阻塞性肺炎的范围不再作为T分期依据

第七版TNM分期将肿瘤导致的部分肺不张或阻塞性肺炎归为T2，若导致全肺不张则归为T3。而在所有研究人群中发现，合并部分肺不张或阻塞性肺炎患者预后与其他因素T2预后一致，但合并全肺不张或阻塞性肺炎患者预后明显好于其他因素T3，因此新版分期无论肺不张或阻塞性肺炎范围大小、累及全肺与否均归为T2。

#### 3.1.4 侵犯膈肌及纵隔胸膜的T分期调整

第七版TNM分期将肿瘤直接侵犯膈肌及纵隔胸膜均归为T3。最新研究发现膈肌浸润患者要比其他pT3患者预后更差，类似于pT4患者，因此新版TNM分期将侵犯膈肌归为T4。对于纵隔胸膜浸润，研究者认为需要进行手术切除或胸腔镜活检后才能进一步确认，和壁层胸膜不同，纵隔胸膜受累没有明显征象，当发现纵隔胸膜受累时往往肿瘤已越过胸膜侵犯到胸膜内组织或脏器，而且病理界定有一定困难，在病理分期中极少见仅单独纵隔胸膜受侵而没有浸润到纵隔内组织的情况，因此将纵隔胸膜浸润纳入临床分期并不可靠，故而在新版分期中删除了纵隔胸膜受累的T分期因素。

### 3.2 关于N分期—增加了病理亚分期

由于以往不同N分期之间生存率差异已经能够很好地反映肺癌患者分期与预后的关系，因此新版分期建议继续沿用原来第七版N分期。但研究却发现对于同一级别的N分期中，临床分期与病理分期生存率差异较大^[3]^，而病理分期往往更能够反映真实的分期情况，研究发现淋巴结转移站数及是否存在跳跃性转移对预后会产生重要影响，伴有多站转移及存在跳跃性转移患者预后明显变差，因此推荐将原来的N1细分为N1a（单站转移）和N1b（多站转移）；N2分为N2a1（无N1转移，直接跳跃到N2的淋巴结）、N2a2（有N1淋巴结转移，同时发生单站N2淋巴结转移）和N2b（多站N2淋巴结转移）。

### 3.3 M分期调整—将寡转移引入肺癌分期

新版们M分期对第七版的M1b进行了较大调整，使之更加细化，与第七版分期最大区别在于引入了远处寡转移病例，其研究结果主要来自西德癌症医学中心Eberhardt等的研究^[4]^。他们对225例单一远处器官出现的单一转移病灶、229例单一远处器官出现的多发转移病灶以及247例远处多个器官出现的多发转移三组患者进行预后分析，发现远处单个器官的单发转移组中位生存时间为11.4个月，明显好于其余两组的6.3个月，显示转移灶数目与患者预后密切相关，而且转移灶数目比转移器官数更有预后价值。因此新版分期将转移器官及转移灶数目纳入分期系统，七版的M1b重新调整为M1b（单个远处器官的单发转移，即寡转移）和M1c（单个器官多发转移或多个器官多发转移）。对于M1a，由于研究发现胸腔内单发转移与多发转移预后无统计学差异，因此仍然沿用原来的M1a分期。新的TNM分期中M1b的预后与M1a类似，明显优于M1c。

### 3.4 TNM分期更加细化

新版TNM分期将原来的Ⅰa期进一步细分为Ⅰa1、Ⅰa2及Ⅰa3期，T1a, bN1由Ⅱa期改为Ⅱb期；T3N1由Ⅱb期改为Ⅲa期；T3N2由Ⅲa期改为Ⅲb期；T3-4N3更新为Ⅲc期；M1a和M1b更新为Ⅳa，M1c更新为Ⅳb，相对更复杂更细致的临床分期使判断预后更加准确，对选择合理的个体化治疗更有针对性。

总之，修订后的TNM分期能够更好的显示患者的预后，在当前精准医学理念的大背景下，新分期标准使肺癌的诊断、治疗以及预后判断更加精准。

## 4 新版TNM分期的局限性

虽然第八版TNM分期较第七版更加全面，能够更好的反映患者的预后，但仍然存在一些问题。

### 4.1 新版分期数据采集的局限性

新版分期虽然增加了亚洲人群比例，但主要为日本病例，中国作为肺癌大国，病例数较少，而且主要为上海和广东病例，不具备代表性。另外虽然首次将南美病例纳入研究，但仍然缺乏非洲、俄罗斯及印度患者的数据。同时由于欧亚人种的个体差异性较大，对治疗的反应及耐受性存在一定差异，其生存率也受到一定影响，例如研究发现对于pN0分期的患者，5年生存率就存在明显的地域性差异，亚洲患者预后最好，5年生存率高达79%，而欧洲患者预后最差，仅为54%，两组之间相差了25个百分点，然而新的分期并没有考虑到人群特征及地域性差异，也没有进行人群特征校正分析，更没有在本次N分期中体现，虽然这种差异随着pN分期的增加而最终消失，但是对于不同地域患者生存率及预后判断可能存在一定偏差。

### 4.2 肺癌驱动基因状态及肺癌分子分型并未在新分期中体现

近年来肺癌分子遗传学研究取得了明显进展，基于遗传特征的分子分型广泛应用于临床，使中晚期肺癌的治疗步入了个体化分子靶向治疗时代，大大改善了部分中晚期肺癌患者的预后，提高了患者远期生存率。然而体现靶向治疗敏感性的肺癌驱动基因状态，如表皮生长因子受体（epidermal growth factor receptor, *EGFR*）、渐变性淋巴瘤激酶（anaplastic lymphoma kinase, *ALK*）及c-ros原癌基因1酪氨酸激酶（c-ros oncogene 1 receptor tyrosine kinase, *ROS1*），程序性死亡分子1（programmed death 1, *PD-1*）表达水平等分子生物学标志均未在第八版分期中有所体现。

### 4.3 新的TNM分期不能对现有的治疗产生改变

尽管目前的对于肺癌的治疗，主要是建立在分期的基础上的，但是改变目前已有的治疗体系，一定要建立在前瞻性的临床研究的基础上。因此可以针对新的TNM分期所产生变化的部分进行前瞻性的临床研究，以制定更好的治疗方案。

总之，新版第八版分期变更的最主要内容是对于T分期的变化，更加细化了肿瘤大小对于预后指导的重要性，与第七版相比有了明显的改善和提高，更能够适应目前临床的需求，更好地反映了不同分期肺癌患者的预后。
